# Western College of Dental Surgeons

**Published:** 1882-05

**Authors:** 


### WESTERN COLLEGE OF DENTAL SURGEONS.

The annual commencement exercises.

The number of matriculates for the session was six.

The degree of D.D.S. was conferred on the following graduates:

| NAME. | RESIDENCE. | NAME. | RESIDENCE. |
|---|---|---|---|
| Wordamon B. Courtney | South Carolina. | Julia C. Mann, | Illinois. |
| | | Louise E. Ottofy | Missouri. |

